# Coexisting cancer regimes: Transformations of breast and lung cancer in the United Kingdom

**DOI:** 10.1177/13634593231176979

**Published:** 2023-05-22

**Authors:** Cinzia Greco

**Affiliations:** University of Manchester, UK

**Keywords:** breast cancer, clinical experiences, disease regimes, innovation, lung cancer

## Abstract

Using in-depth interviews with medical professionals working in the UK, I explore the coexistence of two different cancer regimes in which the different innovations for breast and lung cancer can be located. Breast cancer treatment has seen a protracted series of significant innovations in the context of an emphasis on screening that coexists with a segmentation in subtypes that has allowed targeted therapies for most patients. Lung cancer has also seen the introduction of targeted therapies; however, these can only be used for small groups of patients. Consequently, some interviewees working on lung cancer have expressed a stronger focus on increasing the number of patients undergoing surgery, as well as introducing screening also for lung cancer. As a result, a cancer regime based on the promises of targeted therapies coexists with a more traditional approach that focuses on diagnosing and treating cancers in their early stages.

In this article, I analyse the different forms of medical innovation witnessed by contemporary approaches to breast and lung cancer treatment, using in-depth interviews with medical professionals working in the UK to understand the impact of these innovations in the clinical context. My aim is to show how innovation in cancer care can coalesce around different regimes (Klawiter, 2008), how multiple regimes can coexist in a given moment, and how their development depends on the local organisation of healthcare and on local medical cultures.

Medical innovation has followed different trajectories in breast and lung cancer, to the point that one can identify two disease regimes, one based on the segmentation of disease and targeted therapies, and another based on early detection and organisational innovation. Furthermore, given the incremental nature of much innovation and the existence of plateaus, medical professionals can perceive certain innovation trajectories as unsatisfactory, prompting them to orient their efforts towards implementing other strategies and allowing the coexistence of two disease regimes.

As introduced by Klawiter (2008), the concept of disease regimes describes institutionalised practices and authoritative discourses that construct specific subjectivities. In this sense, a regime is constituted by not only technology and its material impact but also by both the economies of hope that medical discourse builds around technology (Delvecchio Good, 2001) and how specific groups of patients are targeted by innovation and the discourse surrounding it. While Klawiter focuses the concept on the transformations within breast cancer linked to the introduction of mammography, in this article I expand the analysis to the broader range of treatments for both breast and lung cancer to suggest that different regimes can coexist, with the shortcomings of one regime creating space for the emergence or the return of a concurrent regime.

As Delvecchio Good (2001) has observed, proponents of innovations build economies of hope that might overstate the potential impact of an innovation; at the same time, the medical literature also shows signs of work to contain enthusiasm about innovations (Will, 2010). Meanwhile, the review of the sociology of cancer by Kerr et al. (2018) has shown how the redefinition of research and the clinic, the involvement of patients, and the regulation of cancer treatments have been the three main approaches to the social study of medical innovation in oncology. Timmermans and Berg (2003) have highlighted two problematic approaches within the study of medical technology: technological determinism, assuming technology to be an independent variable that influences society unidirectionally, and social essentialism, treating technology as lacking any material impact and as a simple placeholder for other social relations. A further methodological issue prevalent in the study of innovation is the tendency to take one or a few technologies as the starting point. Whether the research looks at an innovation’s diffusion, implementation, costs, or political conflict, there exists a risk of focusing exclusively on more successful or more prominent innovations, omitting the minor, unsuccessful or low impact innovations that Timmermans and Berg (2003) invited to study.

This article’s starting point is not an individual innovation but the clinical experience, which I use to understand the different impacts of innovations on everyday clinical practice (for a recent study adopting a similar approach, see Day et al., 2017). In this way, I explore a range of innovations, taking a broader view that enables me to consider less exceptional or impactful innovations and blind alleys and understand the interactions between different new therapies.

The history of cancer research has notably witnessed the failure of a major programme of innovation, the ‘war on cancer’ declared in the US by then-President Nixon in 1971. The unprecedented investment in and reorganisation of cancer research aimed to find a single cure for cancer, pursuing avenues such as developing vaccines against cancer (cf. Scheffler, 2019). While improvement was obtained for a few cancers, notably childhood leukaemia, the ‘war on cancer’ was recognised as having failed to reduce mortality (e.g. Bailar and Smith, 1986); since then, the consensus has shifted on considering a single cure for all cancers improbable. This has not stopped governments from launching new initiatives to try and tackle cancer through concentrated investments in a short period of time, but even the US ‘Cancer Moonshot’ launched in 2016 (the closest equivalent of the ‘war on cancer’) has stopped short of searching for a single cure for all cancers.

The failure in finding a cure has intensified a regime focused on early detection and interweaved with a ‘cancer schemata’ that developed in the early 20th century: a vision of cancer in which ‘small, localized tumors were an early stage in the development of a malignancy’ (Löwy, 2010: 2) and were considered curable. That is, having abandoned the project of finding a cure for all cancers, the goal of diagnosing cancer early became even more central. Although this vision simplifies a more complex biological reality, it still predominates the way cancer risk is managed (cf. Löwy, 2010) and it is at the origin of cancer screening programmes.

Alongside early diagnosis, the structuration of clinical trials as a research approach and the development of new pharmacological treatments has been central to the establishment of oncology as a medical speciality since the 1950s (Keating and Cambrosio, 2012). More recently, genomics has become central to innovating cancer treatment, which is increasingly oriented towards segmenting cancer into increasingly small subtypes and identifying biomarkers for targeted therapies (cf. e.g. Kerr et al., 2021). Such developments are linked to a second cancer regime, involving promises of ‘personalised medicine’, where different patients would receive therapies that are best suited to them, being at the same time more effective and with fewer side effects (cf. e.g. Day et al., 2017; Kerr et al., 2021). However, the concept of personalised medicine has, in turn, been subject to a range of critiques, including that it constructs an economy of promises that are difficult to fulfil, that it excludes groups of patients, and that it risks focusing on costly treatments that benefit a small number of patients while disregarding medical measures that could impact most patients (Day et al., 2017; Kerr et al., 2021; Sturdy, 2017). A recent report on the status of cancer research in Europe has further suggested that discovery research, in particular pharmaceutical research on targeted therapies, is significantly over-emphasised, especially in relation to the need for more implementation research (Lawler et al., 2023).

In this article, I focus on breast cancer and lung cancer, which are among the most common in the UK – in 2017, breast cancer was the most diagnosed and lung cancer the third most diagnosed in England, accounting for 15.1% and 12.7% of all cancer registrations (ONS, 2019). Comparing the two enables the reconstruction of two different histories of innovation: while breast cancer has historically been characterised by high levels of innovation, lung cancer has often been stuck on innovation plateaus, and has recently been identified as the most under-researched cancer in Europe in relation to the overall cancer burden (Lawler et al., 2023). Comparing breast and lung cancer allows to capture differences between major cancers with good and bad prognoses and high and low levels of innovation, but I do not claim that such differences extend, for example, to cancers with particularly bad prognoses (e.g. pancreatic) or to rare cancers.

The history of breast cancer is usually presented as marked by a series of important breakthroughs, including the success of hormonal and targeted therapies and the discovery of the BRCA genes,^1^ which contributed to lengthening survival times (cf. infra), to the extent that early-stage breast cancer (ESBC) – a cancer that has not spread to other organs – is now defined as ‘curable’. It is also one of the types of cancer around which the rhetoric of early diagnosis and the importance of screening were built. Moreover, breast cancer is considered the quintessential female cancer; not only does it affect the breasts, but it is also associated with a glamorous and sexy image often adopted by awareness campaigns (cf. King, 2008; Sulik, 2010).

In contrast, lung cancer has not only historically been strongly associated with smoking, and thus perceived as a ‘male disease’, it has also witnessed scarce innovation and limited changes in survival times (cf. infra). Lung cancer has been described as ‘recalcitrant disease’ and its history is characterised by scarce innovation and limited success in improving survival times, with the stigma linked to smoking as one explanation for the limited resources historically allocated to lung cancer research (Timmermann, 2013). Screening for lung cancer has been contemplated since the 1940s, with plans to use the infrastructure already in place for detecting tuberculosis. However, doubts about the real benefits for patients in terms of lengthening survival time and the gradual dismantling of the infrastructure itself meant that such programmes were never implemented. The introduction of a new detection technique in the 1990s – the computerised tomography (CT) scan – reopened the debate about lung cancer screening, especially its utility and cost-effectiveness (see Timmermann, 2013 for a comprehensive history). The clinical experiences and perceptions that I present in this article are closely linked to these debates.

## Methodology

The present analysis is based on in-depth interviews with medical professionals working on breast and lung cancer. Among these participating interviewees, there are 15 oncologists (including clinical oncologists, who administer radiotherapy in the UK), 12 nurses, 5 respiratory physicians (who have a key role in lung cancer treatment in the UK), 2 surgeons and 2 radiologists, along with 1 biologist and 2 administrative personnel of NHS trusts. The main group of interviews derives from research that I conducted between 2019 and 2020 in Greater Manchester and Cheshire. That research examined the impact of medical innovations and healthcare policy change on the experience of patients with breast and lung cancer and included interviews with 23 medical professionals. Additionally, for discussions on breast cancer in particular, I also use interviews collected as part of research conducted between 2017 and 2018 on transformations of metastatic breast cancer (MBC). For that research, I conducted 16 interviews with medical professionals working on MBC in Greater Manchester and other areas of Northern England.

In both cases, the interviewees were contacted either directly by email or through a snowball procedure. The researcher is a social scientist with no specific links with the interviewees. The interviews focused on the participants’ experiences treating breast or lung cancer from the beginning of their careers to the present day. The interview guide included participant backgrounds, what they considered the main changes in the treatment of lung or breast cancer, how they had seen the experiences of patients change over the years and their perspective on the future development of the field. Additionally, participants in the more recent research were also asked about which changes in healthcare policy they considered to have impacted their work, and participants in the earlier research on MBC were also asked whether MBC could be considered a chronic disease.

This approach has provided historical insights into the development of treatments for breast and lung cancer, with a depth generally aligned with the length of interviewee careers (although a few interviewees did discuss the earlier history of the conditions). Furthermore, this approach enabled the identification of the relevance of different innovations through the perspective of the clinical experience of the individual interviewees.

Most interviews were recorded; in the few cases in which the interviewee preferred not to be recorded, I took notes during and after the interview. The interviews were analysed through a systematic internal and cross-interview analysis to identify both recurring themes and deviations, and, following the extended case method (Burawoy, 1991), the ethnographic data were put in relation with structural dimensions studied through medical literature and policy document, and compared systematically with the existing theories to identify points of divergence. Both research projects received ethics authorisation from the NHS, and each interviewee gave verbal and written consent to participate in the research. All the names used for the interviewees are pseudonyms.

## The political landscape of innovation: The NHS, the Manchester devolution and cancer pathways

The success of biomedical and technological innovations is influenced by social, administrative and political contexts. While biomedicine tends towards standardisation, the way in which therapies are administered depend on the local availability of the most recent technologies, on local biomedical cultures, and on the local organisation of healthcare. This applies to more standardised innovations such as new drugs, and more so to innovations that tackle the organisation of healthcare, such as those I discuss in this article for screening and patient pathways. The UK and Greater Manchester constitute an interesting vantage point from which to analyse these intersections. The NHS is the prototype of a public, tax-funded and free at the point of use healthcare system that aims to offer all citizens the same level of quality of care. However, there has been considerable discussion about territorial variations in the availability and quality of healthcare services, a phenomenon described as ‘postcode lottery’ (Bungay, 2005). Of course, the UK is not the only country experiencing geographic variation in healthcare quality; however, unlike other countries (see Greco, 2019 for the Italian case), internal health-related mobility is less common in the UK. Over the years, different national governments have implemented policies to standardise healthcare services across the country, aiming to address the postcode lottery (see e.g. Bungay, 2005). In this context, Greater Manchester in particular has seen an important policy change. In 2017 Manchester was among the ten areas in the UK with the lowest life expectancy (Purdam, 2017). Greater Manchester has obtained and implemented a devolution (‘Devo Manc’) that significantly transferred decision-making power (and money) in several key areas, including health and social care responsibilities, from the national government (Coleman et al., 2015). While generally presented as a positive change (see e.g. Vize, 2016), the Devo Manc has also been criticised. For example, the local branch of the group Keep Our NHS Public has presented the project as a reorganisation plan that includes cuts to staff and services across the region and could open the door to forms of privatisation of the services.^2^

The cancer care landscape is also consequently changing, with the medical professionals interviewed expressing a generally positive view of the transformations prompted by the Devo Manc, seeing in it an occasion for more flexibility. According to one interviewee, Dr Marlon, ‘the Devolution, it allows us opportunities’.

As a result of the devolution, the region has also established Greater Manchester Cancer (GMC), a programme that supervises the organisation of cancer care in Greater Manchester and Eastern Cheshire.^3^ One of GMC’s aims is to improve the quality of pathways for cancer patients from screening to follow-up care. Pathways, both as protocol that standardises treatments and as ways of organising patients’ movements between different services and institutions, have been developed extensively in various healthcare contexts. Research on pathways has raised criticism linked to accountability, as in some contexts patients can be treated in variable ways, and the pathway documentation become additional work to create a standardisation of treatment that is mostly on paper (Allen, 2012; Håland and Melby, 2023). At the same time, the standardisation of pathways itself has been criticised, as it can fail to consider the complexity, difficulty and unpredictability of illness, particularly in the case of cancer (Llewellyn et al., 2018). According to a consultant interviewed in my research, the role of pathway boards is to standardise cancer care in Greater Manchester and reduce the risk of a postcode lottery. However, for cancer, and lung cancer in particular, a swift arrival to diagnosis and treatment is considered even more important than standardisation (see infra).

In my fieldwork, the role of the Devo Manc was rarely mentioned by medical professionals working in the breast cancer field, potentially because breast cancer’s clinical pathways seem, to a certain extent, clearer and more well-established. However, Devo Manc became central to many interviews with lung cancer professionals, particularly those working at implementing new screening programmes. Several interviewees underscored how, for lung cancer, the condition is usually diagnosed late – systematic screening is not available, and most symptoms masquerade as chest infections – and that delaying the passage from GP referral to diagnosis can mean a stage shift in the tumour. Thus, substantial efforts have gone in streamlining the passages from the GP to treatment (see infra). Most medical professionals interviewed see a more flexible system as permeable to more effective changes and try to use it to advance their ideas of what constitutes a useful and beneficial medical innovation.

## Breast cancer: A mosaic of treatments for a fragmented disease

At the centre of one of the largest cause-related marketing campaigns and associated with the pink ribbon symbol (Klawiter, 2008), breast cancer is probably the most well-known cancer, to the extent that it influences the experience of patients suffering from other cancers (Bell, 2014). Breast cancer is also considered a success story in oncology, presented as a disease that can be not only treated but even cured. The therapeutic success of the disease has been linked to the rapidity with which it is diagnosed.

Two of the interviewees emphasised the role of Patrick Forrest for breast cancer treatment in the UK; Forrest, a surgeon, produced a report that led to the introduction, in 1987, of a breast cancer screening programme aimed at women over 50 (see also Griffiths et al., 2010). Criticism of the disease regime linked to breast cancer screening has mostly focused on two points: (1) it is sometimes falsely presented as prevention, rather than early diagnosis, which improves treatment but cannot exclude relapse or metastasis (Klawiter, 2008; Sulik, 2010), and (2) it can cause overdiagnosis, subjecting patients with tumours that would never have become problematic to unnecessary treatment that causes damage potentially outweighing the benefits of early treatment (Carter, 2021; Sulik, 2010). The only interviewee to mention a downside to breast cancer screening, Dr Arthur, focused however on the increased number of women requesting breast checks outside of organised programmes, suggesting that this phenomenon, known as ‘opportunistic screening’, risks ‘overrunning’, as he said, ‘the system’, and suggested that GPs should more carefully select the women referred for breast cancer screening. Such an approach would mean that the subjectivity linked to breast cancer screening would be mostly limited to the women in the targeted age group. However, self-initiated screening is both a proof of the importance public discourse places on breast awareness (compare Griffiths et al., 2010), as well as a form of agency that can usefully detect tumours developing in patients outside of the screening age range (Greco, 2020). Furthermore, the idea of scaling back the number of referrals contradicts the message that women should check their breasts regularly to enable timely intervention if something is wrong.

A complex mosaic of treatments – surgery, chemotherapy, radiotherapy, hormonotherapy and monoclonal antibodies – is currently used against breast cancer; some have been introduced more recently while others have been used for decades. For example, the role of female hormones in the development of breast cancer began being understood in the 1950s (Howell et al., 1997), and, towards the end of the 1970s, the first hormonal drug, tamoxifen, was introduced with the aim of reducing interactions between oestrogen and breast cancer cells. Tamoxifen, discovered by chance, while trying to develop a new oral contraceptive, is considered the first example of a targeted therapy for cancer (Jordan, 2006). It has paved the way for the idea that specific cancer subtypes could be treated with drugs that focused their effect on cancer cells and were less toxic for the organism. The drug’s discovery and approval returned interest to hormonal treatments for cancer at a moment in which much of the attention was focused on chemotherapy. Different types of hormonal drugs have since been developed, such as a class of drugs known as aromatase inhibitors (Eraso, 2020). Another important breakthrough in the treatment of breast cancer has been the introduction of monoclonal antibodies, proteins that attach to specific targets. All of the interviewees working in breast cancer agreed that the introduction of Herceptin, the first monoclonal antibody used for breast cancer, combined with knowledge of how to identify the type of tumours (Her2+) that can benefit from it, has significantly changed the prognosis for a specific group of patients. According to Dr Julia, a consultant whose career began in 1999, ‘all the time I’ve been a consultant I’ve been able to access it [Herceptin] for metastatic patients. And some of those patients who started in the early 2000s are still on it now, and that’s the big surprise’. Herceptin was the first of a set of new drugs based on the same principles. Several monoclonal antibodies are now available, and new treatments combining monoclonal antibodies with additional molecules, such as radioactive particles and chemotherapy drugs, have been developed, providing additional resources for patients, and opening to a cancer regime based on targeted therapies. Almost all of the medical professionals interviewed listed several new treatments available against MBC. The research for MBC also benefits patients with ESBC because, as Dr Thomas explained,

The development of drugs for breast cancer is: usually you start treating someone with advanced breast cancer, and if it looks very good you then test it after surgery for breast cancer, and if it looks very good you then test it for prevention of breast cancer, so that’s the process we’ve done over the years.

This extract is typical not only of the kind of answers that I found in the research, but also of how breast cancer research is presented in the literature. Hormonal treatments and monoclonal antibodies are indeed routinely prescribed to patients with ESBC with the aim of preventing a relapse. That these treatments reduce (without ever eliminating) the risk of a relapse contributes to the perception of ESBC as a curable condition (cf. Greco, 2022b and, for how the possibility of a relapse leaves patients in a liminal state, Rees, 2017). For women with MBC, experimental treatments available through clinical trials are an option when other treatments stop producing results. A significant literature exists concerning inequalities in clinical trial access (e.g. Kerr et al., 2021), and in MBC the complexities of clinical trials add to the uncertainties patients already experience with regard to their life expectations (Greco, 2022a). However, compared to other cancers, MBC seems to have witnessed more successful innovation, to the point that Dr Julia described clinical trials as less relevant for the condition: ‘[when] you got a standard treatment that’s good, you don’t need to do a trial, because you’ve got something that’s really good, it’s going to be second or third line’.

Several consultants, especially those with more experience, underscored how, until a few decades ago, they did not have the ‘molecular information’, as Dr Luke put it, that now guides the practice. According to Dr Brad,

Originally, we didn’t have oestrogen-receptor, progesterone-receptor status. We didn’t have HER2-status. You didn’t have the different subtypes of cancer we now consider as guiding different treatments. So there’s a level of sophistication we use now that we didn’t have before.

This extract was also typical of the answer I had from a number of oncologists, who often presented biomarkers as waiting to be discovered. Such ‘molecular information’ in breast cancer is particularly important because only 10%–20% of patients do not express any of the three main biomarkers (ER, PR and HER2) that enable targeted treatment (Boyle, 2012). Said otherwise, the subjectivities created by targeted treatments in cancer include most women with breast cancer. The current ‘level of sophistication’ mentioned by Dr Brad and the long list of therapies available reinforces the idea that breast cancer therapies and breast cancer itself are tending towards segmentation and hyper-specialisation. Several of the medical professionals interviewed insisted that it was antiquated and incorrect to consider breast cancer a single condition and that it would be more appropriate to tackle it as a group of different diseases that can affect the breast, each with its own genetic and biological characteristics and, more importantly, each with its own set of treatment possibilities. Although this aligns with the promises of targeted therapies and personalised medicine discussed in the introduction, no interviewee mentioned the critiques and limitations that have been advanced in the social science literature around the concept of personalised medicine.

The therapeutic landscape for breast cancer, and especially for MBC, does not include a single treatment able to cure the disease but rather a complex mosaic of options that can keep the cancer at bay for a longer period without ever eliminating it. If ESBC is increasingly defined as curable, MBC is described as ‘treatable but not curable’ and increasingly represented as a chronic disease in the medical literature (Greco, 2022b). Many doctors are of the opinion that this trend will continue, and there will be more therapies available and an increasingly stratified approach, showing important economies of hope (Delvecchio Good, 2001) linked to targeted therapies. However, some medical professionals disagree with the vision of breast cancer as destined towards further stratification and significantly improved survival times. Some consultants, such as Dr Mark, believe that the next decade will see only ‘incremental benefits from endocrine therapy, targeted therapies and new chemotherapies’.

Another rarely mentioned aspect of the impact of new therapies is the ways that their use clashes with the organisation of healthcare facilities. Dr Maria, a breast cancer consultant, acknowledged that:

A challenge for us [is] to get used to different types of treatments with several side effects, with interferences with several other organs, so with Herceptin we learn how to monitor the heart function, we had to remind ourselves how to treat heart failure, we had to engage with cardiologists, but cardiologists are busy people too.

Dr Maria was relatively set apart in referring to the need for other specialities to get involved, as the prevalent discourse focuses on the reduction of side effects and the possibility to administer higher dosage of therapy, with rarer references in the interviews to the need for side effects to be managed by other professionals. She continued by explaining that other treatments that are emerging within the therapeutic landscape can cause serious side effects, including fatal colitis attacks. To support patients, it is necessary to collaborate with gastroenterologists, she said, who, not only, like cardiologists ‘are busy people’, but who may have limited familiarity with specific side effects. The complexity of some new treatments necessitates intensifying collaborations between different medical specialisations and different healthcare professionals. However, it can be difficult to maintain a strong interdisciplinary approach in the treatment of cancer, particularly given the limited available resources and overextended medical professionals.

## Lung cancer and early detection as innovation

Lung cancer is strongly associated with lifestyles described as ‘unhealthy’; many of my interviewees explained its high incidence in Greater Manchester with the low socio-economic profile of some parts of the population and the fact that people in lower social classes are more likely to consume tobacco and alcohol. Being a smoker is notably the main risk factor mentioned by medical professionals, with other possible causes – for example, occupational exposure to toxic substances or air pollution – usually considered less statistically relevant. Lung cancer treatments include the same triad already observed for breast cancer: chemotherapy, radiotherapy and, for some patients, surgery. Targeted therapies are also available for subgroups of patients. However, following the introduction of platinum-based chemotherapy, lung cancer treatment innovations plateaued for several years, with only minor innovations introduced, leading the disease to remain ‘recalcitrant’ (cf. Timmermann, 2013). According to Dr Ken, a respiratory consultant, ‘The standard chemotherapy regimes we use in lung cancer today are pretty much the same as that plateau that was reached in 2002. Things that have changed is the introduction of molecular targeted therapy, and then immunotherapy’. Several consultants insisted that in recent years there have been improvements in the stratification of tumours according to biological markers. Meanwhile, radiotherapy has witnessed significant improvements, as Dr Michel explained:

IMRT [. . .] intensity-modulated radiotherapy, it’s basically achieving a high dose over the tumour and sparing the normal organs as best as possible [. . .]. So that’s a new innovation [that] treats more complex patients more quickly [. . .]. I suppose SABR is also coming through for oligo-metastatic disease; so in other words people who have one or two deposits [. . .] of cancer that spread in another organ, in the past we used to decide [to try] to remove the spots or give them chemotherapy but, you know, they often progress. Now, people have started using a blast of radiotherapy to burn it away. [Radiotherapy] can delay chemotherapy [and] give extra years before [patients] need chemotherapy because you’ve blasted the disease.

Contrary to the extensive references that most interviewees made to drug-based treatments, Dr Michel was one of the few to detail significantly improvements in radiotherapy. Despite these improvements and innovations, there is a general consensus that survival times remain limited and that pharmacological treatments and radiotherapy produce limited results. Dr Ken provided a summary of the current situation:

In broad terms, for all cohorts with lung cancer, the median survival remains poor: twelve, thirteen months. You know, small groups get much better survival, but it doesn’t change the overall statistics for the whole population. So, despite all of the advances in drug therapy, the median survival for lung cancer hasn’t changed in ten years.

Dr Ken was part of a minority of medical professionals working on lung cancer who was openly pessimistic about drug-based improvements in lung cancer, and one should also note the reference to median survival time as a measure of improvement that explicitly acknowledges improvements when these apply to at least half of the patients. When I asked how many patients present biomarkers that allow for targeted treatment, Dr Ken indicated ‘around 10%’, and added: ‘it’s a small step forward for that small cohort, it’s not that helpful for the majority of patients with non-surgically-treated lung cancer’. He had a more optimistic view of immunotherapy, considering it ‘probably a very big change’ because it is ‘a little bit more broadly applicable to people who have smoked and developed lung cancer’.

To expand on Dr Ken’s observation, not only have the 15%–20% of patients with small-cell lung cancer witnessed extremely limited treatment innovations, but patients with non-small-cell lung cancer have seen innovations that often only apply to small subgroups. In the case of lung cancer, the main biomarker for which targeted treatments are available is EGFR, a biomarker with widely variable incidence, being as high as 47% in East Asia, but as low as 15% in Europe (Midha et al., 2015). The second most common lung cancer biomarker for which targeted treatments are available, ALK, is present in 3%–7% of the patients, with the remaining biomarkers for which treatments have been identified all representing under 5% of patients with lung cancer (Halliday et al., 2019). Thus, it is clear that targeted lung cancer therapies can serve a significantly smaller percentage of patients when compared to the situation in breast cancer. Immunotherapy can be used for a larger number of patients, the main indication being expressing the PD-L1 biomarker in more than 50% of tumoral cells, but some literature has observed that PD-L1 is an imperfect biomarker and that immunotherapy only produces a response in the tumours of a minority of patients for whom it is indicated (Doroshow et al., 2019). In sum, the subjectivities created by targeted therapies in lung cancer exclude large groups of patients.

Given these difficulties treating lung cancer, even with the development of significant therapeutic innovations, some of the doctors I met indicated other possible ways to improve survival times. Among the strategies mentioned, efforts to increase the number of patients undergoing lung surgery was presented as promising. While surgery for breast cancer is routinely performed, in particular for ESBC patients, lung cancer surgery is more complex and can currently be performed in only a small number of cases in which the tumour is small enough to intervene. However, according to Dr Horace, a lung cancer physician, in recent years, there has been ‘a huge change in terms of treatment, so surgery has changed, there’s now minimally invasive surgery, called VATS [video-assisted thoracic surgery]’. Dr Ken explained further:

Thoracic surgery has become more accessible – the quality of surgeons, intensive care, surgical techniques – mean[ing] that you can do a lot more to a lot more co-morbid patients [. . .]. In broad terms, you can operate on people who are quite sick – they don’t have to be very fit – so we’ve got a way of accommodating that. And we have been very aggressive in pushing the surgical agenda, so that the resection rates now, you know, across the country they might be 17%, ten years ago they might’ve been 10%; in this institution we have always been high, 30% of surgical resection rates, so we push the surgical agenda and the physiological challenge and the assessment.

From the extract it is clear how Dr Ken’s description unites the advancements in surgery in general with the specific initiatives taken in his own institution. While Dr Ken was probably the most explicitly pessimistic among the interviewees about targeted treatments, a number of other interviewees focused on non-pharmaceutical improvements for lung cancer. For this cancer, the relevance of surgery and the emphasis on early diagnosis are intertwined. According to Dr Marlon,

One of the big problems with lung cancer globally is delayed presentation [. . .]. People with early-stage lung cancer, when it’s curable, they have zero symptoms, we don’t know they’ve got it, and by the time they’ve [got] symptoms, it’s stage III–IV, and it’s more difficult to treat. So, obviously, one of the topical things is screening.

Dr Horace, after discussing the new treatments available for patients with lung cancer, added:

And I have to say, all of the changes that have come in, they do not really make a massive difference to the patient in terms of survival. The main one is actually diagnosing them earlier, so the critical, critical step is finding the cancer early, and that’s the thing that actually is the drive for survival.

Dr Oscar, a lung cancer physician with several decades of experience and a strong interest in early lung cancer screening, was even more explicit in this regard:

We’re still accepting that patients with lung cancer mostly are going to be identified when they’re incurable. And yet, not only it is preventable, it’s also predictable, on risk scores, you know, is very, very predictable. So we should have been screening for lung cancer years ago. It’s the most predictable cancer out there, you know, you can look at the population and calculate precisely calibrated risk scores.

The three doctors – Marlon, Horace and Oscar – present different points about the role of screening: the fact that it has more potential than pharmaceutical therapies (Horace), the importance of treating before the cancer advances and symptoms appear (Marlon and Oscar), and the historical delay in attempting to screen for lung cancer (Oscar). It should also be noted that these similar discourses were presented in different terms, with Dr Oscar being more senior and highly critical of the previous organisation of healthcare, and Dr Marlon more junior, optimistic and focused on the details of introducing a screening programme. Despite progress enabling a larger number of patients to access lung surgery, to maximise the positive impact of lung surgery, it is important to detect smaller tumours in asymptomatic patients, necessitating the extension of screening programmes. Greater Manchester is described as a ‘progressive’ region that is not only addressing the high lung cancer mortality that has historically affected the area but is doing so by developing pilot programmes that can be implemented on a national scale. In addition to the regional pathways already discussed, some trusts have designed localised solutions to streamline and expedite the diagnostic process, allowing patients with suspected lung cancer to receive a CT scan within a day (for an exploration of a local pathway, see Evison et al., 2020). Greater Manchester has seen the trialling of a new screening programme for lung cancer involving high-risk patients being invited to participate in a lung health check at mobile units positioned in specific areas of the city considered to be at higher risk (see e.g. Crosbie et al., 2019). The programme has been described as an innovative approach that could serve as a pilot for a broader screening policy. One interviewee, Dr Marlon, was involved in the project and provided a detailed explanation of the rationale behind the programme design, being the interviewee that went most into detail:

Essentially, it’s things like travel, fear of hospitals, you know, cost, these are all issues why these deprived populations, for example, wouldn’t come, would need to take two buses to get to your hospital, to undergo screenings. So, that’s why we designed the program. And the idea in what we did was we provided lung health checks rather than lung cancer screening. And the reason for that was speaking to patients about lung health, that’s less scary [. . .]. Another thing is we have mobile trucks, instead of asking them to come here, we went to deprived areas of Manchester, supermarket car parks etcetera. So very accessible, very friendly, welcoming [. . .]. So we definitely managed to reach the most high-risk people, we did the health check, and within that, we used a lung cancer risk score to determine who’s at high risk of cancer. So not everybody that had a health check got a CT scan [. . .]. So we only scanned those at high risk, and we found lots of cancers, so we, you know, had 4.4% within a year have cancer, 80% of them are early stage, most of them had curative treatment.

Pharmaceutical innovations for lung cancer are delivering some relief to patients, but their impact remains slow and limited. Within this context, an active group of medical professionals is redefining the idea of medical innovation. In this case, identifying cancer cases as soon as possible is the main element and is based not on ‘new techniques’ but on a new, large-scale way of implementing existing screening techniques. Many medical professionals have insisted that these measures are not only more effective, but also more beneficial in terms of cost-effectiveness. The possibility of developing pilot programmes, such as that described by Dr Marlon, is further enabled by the greater autonomy of Greater Manchester in relation to the national government.

## Conclusions

In this article, I have analysed the coexistence of two cancer regimes in which the different innovation for breast and lung cancer can be located, exploring the interplay between early diagnosis and targeted therapies. The analysis shows how the concept of cancer regimes can be used to understand the co-presence of different lines of innovation that rely both on different ideas about cancer and possible treatments (e.g. targeted therapies or early diagnosis) and on discourses about how cancer survival will be improved in the future. Further, it shows how medical innovation, and particular innovation based on healthcare organisation, are shaped in different ways by local contexts, in this case being filtered through the organisation of the NHS and the local specificities of the Manchester Devolution.

A personalised approach and segmentation into several subgroups have characterised breast cancer, with most patients now having access to targeted treatments. These medical innovations have changed the nature of the disease and intensified the gap between ESBC and MBC, with the former now generally considered a curable disease and the latter a treatable disease that can be held at bay by a complex arsenal of therapies. They have also created new subjectivities for patients around the presence – or absence – of specific markers and alimented new hopes and expectations around breast cancer. According to some medical professionals, such as Dr Mark, the breast cancer innovation trajectory has reached its peak; meanwhile, for others, such as Dr Maria, new therapies have introduced disruptions into the clinical routine.

The segmentation of subtypes of cancer according to the biomarker model already seen for breast cancer is also becoming important for lung cancer. However, some doctors remain sceptical about such pharmacological advances, noting that most can only be used for small groups of patients, leading them to reiterate the importance of early diagnosis. As discussed, screening programmes for breast cancer have existed in the UK since 1987, while there have never been early screening programmes for lung cancer. Most of the doctors interviewed strongly insisted that the lack of such screening programmes harms patients, especially in the most vulnerable segments of the population, namely, working-class people, among whom smoking rates are higher. None of the interviewees working on lung cancer mentioned the limitations or downsides of screening (Carter, 2021) or the fact that there have already been discussions in the past about the possibility of conducting lung cancer screening programmes (Timmermann, 2013). Instead, they insisted on the novelty of the proposal, especially the pilot study conducted in Greater Manchester, rendering the flexibility enabled by the Devo Manc as positive.

Another development within lung cancer is increased attention on surgery. Breast cancer was among the first cancers to be treated surgically; given the breast is a non-vital organ, almost all patients with ESBC, and some patients with MBC, can be offered surgery. However, the more challenging location of lung cancer tumours has meant that, despite a history of attempting to increase the rate of patients undergoing surgery (Timmermann, 2013), it has remained an option for only a limited number of patients. Some of the interviewees emphasised that innovations in the field of lung cancer surgery are changing this situation, with improved techniques allowing to operate older patients with more co-morbidities. This opportunity was also presented as strictly linked to the possibility of detecting smaller, operable tumours through screening and access to specific medical contexts featuring surgeons specialised in performing this type of surgery.

Furthermore, comparing the trajectory of innovation of breast and lung cancer reveals that patients with breast cancer, whether ESBC or MBC, receive a mosaic of therapies – surgery, radiotherapy, chemotherapy, hormonotherapy, monoclonal antibodies – some of which continue for several years, including in the case of ESBC. In contrast, for patients with lung cancer, being able to receive surgery appears the main factor, and it is not usually accompanied by drug therapies. Although, overall, lung cancer has a more negative prognosis than breast cancer, survival is most often linked not to the possibility of using different therapies in sequence but to the success of a single therapy, namely, surgery. That is, while pharmaceutical innovations for breast cancer have produced a complex landscape of treatments for the disease, in the case of lung cancer the aim is to propose to patients a modern version of early diagnosis and surgery, which can enable operation on small tumours. It seems that the main goal of innovation for lung cancer is to create a new entity, early-stage lung cancer, that mirrors ESBC and symbolises the hope for cures because it has been ‘caught in time’.

The trajectory of innovations of lung cancer shows the extension of the early diagnosis regime. The targeted therapies regime is less successful in lung cancer than in breast cancer, or at least not successful enough to create a narrative of lung cancer as a ‘curable’ condition. Thus, medical professionals have extended the idea of early detection and organised screening, the importance of which has never been supplanted by personalised medicine, to new types of cancer. Accordingly, it is worth speculating whether lung cancer screening will become the unstable and controversial tool that breast cancer screening has become, marked by tensions between organised and ‘opportunistic’ screening and controversy around its utility.

Promises based on medical innovation have been shown to be particularly relevant in the discourse of oncology and to shape the experiences of patients (Delvecchio Good, 2001), and further to push normative models on patients with breast cancer in particular (King, 2008; Klawiter, 2008; Sulik, 2010). Identifying different cancer regimes allows not only to understand the trajectories taken by innovation in cancer, but also to reconstruct a significant part of the promises and models of behaviour proposed to patients with cancer. Analysing cancer regimes also allows to show why popular research directions, such as targeted therapies, can be emphasised even in cases in which the results are relatively modest, and the way in which other approaches to innovation (e.g. lung cancer screening) can emerge as an alternative. As mentioned in the introduction, comparing breast and lung cancer allows to capture some of the main dichotomies for major cancers (good/bad prognosis, high/low innovation, screening/targeted therapies), but further research in the variety of regimes existing for other cancers could help better understand the workings of oncology and its promises.

The two cancer regimes that my research identified represent the contrast between the ‘mass’ nature of early diagnosis and organised screening – which involves all healthy individuals pertaining to specific categories – and the segmented nature of targeted therapies, which include dubious promises of ‘personalised’ medicine. Furthermore, comparing breast and lung cancer shows how treatments and innovations are articulated around new and old interpretative frames within biomedicine and how medical professionals’ willingness to promote certain forms of innovation adapts to and modifies health system management.

## Notes

1. There is an extensive literature on genomics and diagnosis in breast cancer on which I do not focus here, including research considering BRCA genes that predispose to breast cancer and the creation of pre-vivors (e.g. Gibbon et al., 2014; Löwy, 2010; Werner-Lin et al., 2012) and the use of Oncotype DX for determining the risk of relapse and the utility of chemotherapy (e.g. Kerr et al., 2021).

2.
https://keepournhspublicgmcr.com/2015/03/08/the-true-purpose-of-the-greater-manchester-nhs-devolution/

3.
https://gmcancer.org.uk/about/
